# Supplementary material for: Reaction-induced rheological weakening enables oceanic plate subduction
Source: Nat Commun. 2016 Aug 26;7:12550. doi: 10.1038/ncomms12550 (PMC5007449; doi:10.1038/ncomms12550)
Supplement: Supplementary Information — Supplementary Figures 1-4 [file ncomms12550-s1.pdf]

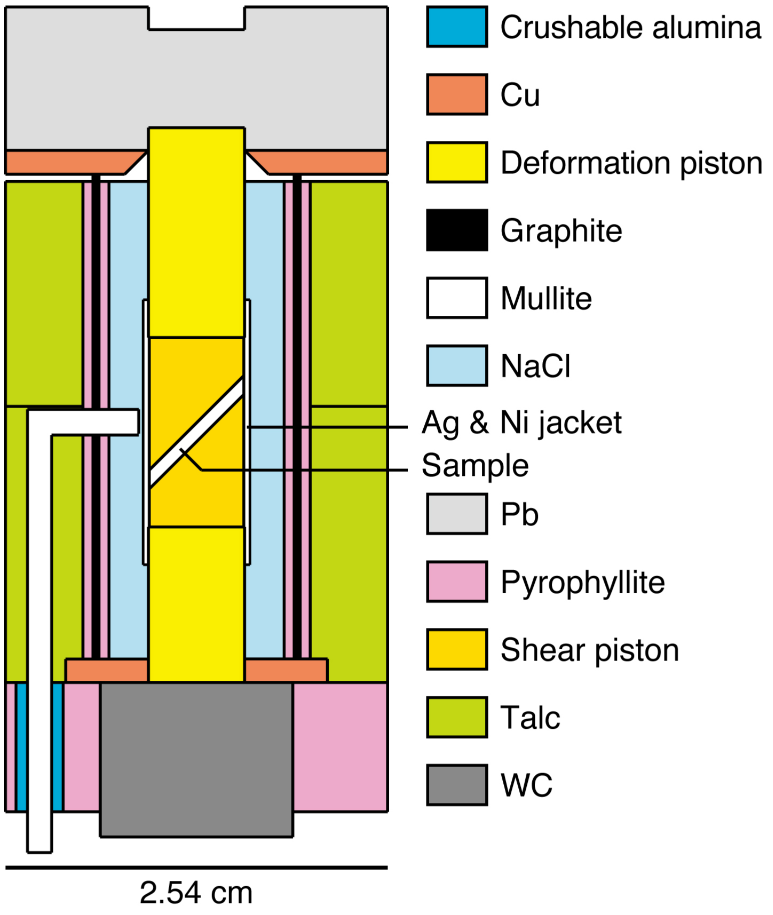

Supplementary Figure 1. Schematic cross-section of the sample assembly for simple-shear deformation experiments using a Griggs-type apparatus. The sample was sandwiched between alumina pistons that were cut at 45° from the direction of maximum compression.

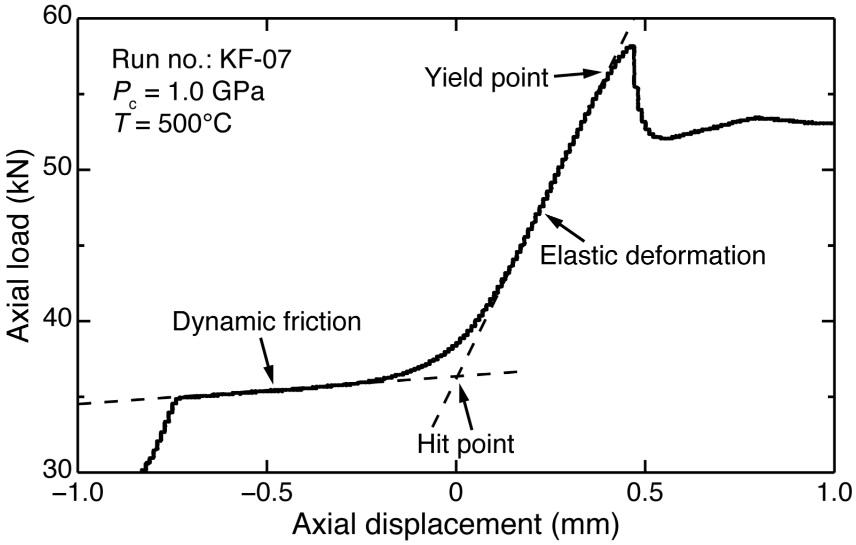

Supplementary Figure 2. Typical axial load–axial displacement curve for an olivine–orthopyroxene sample. The sample (Run KF-07) was deformed at an axial displacement rate of 1.9 × 10^{-5} mm s^{-1}. The hit point is the intersection of the extrapolated trend lines for dynamic friction and elastic deformation.

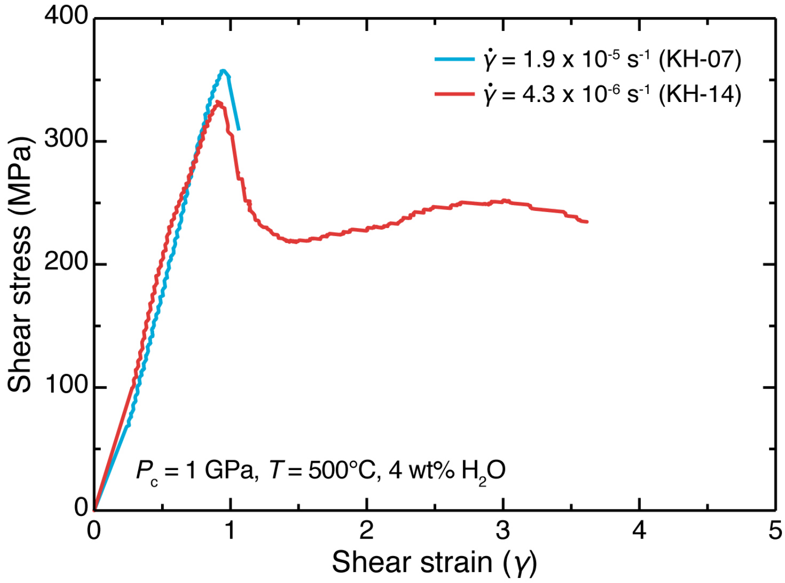

Supplementary Figure 3. Shear stress–shear strain curves for an olivine–orthopyroxene (Run KF-07) and orthopyroxene (Run KF-14) samples. The samples were sheared at a confining pressure of 1 GPa, a temperature of 500°C, and different shear strain rates ($\dot{\gamma}$), under hydrothermal conditions.

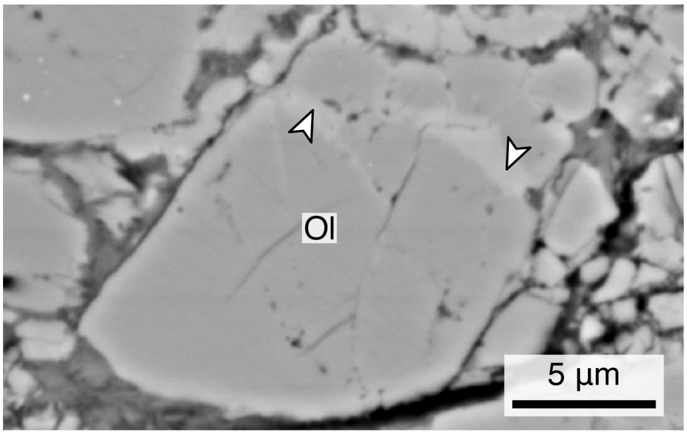

Supplementary Figure 4. Back-scattered electron image of a deformed olivine–orthopyroxene sample. Run KF-07 (heating time: 48 h). Note an olivine (ol) grain characterized by iron-rich rims (indicated by small arrows).
